# Is adenosine deaminase (ADA) activity in saliva and serum a more accurate disease detection tool than traditional redox balance parameters in early-lactating dairy cows?

**DOI:** 10.1007/s11259-023-10069-2

**Published:** 2023-01-06

**Authors:** Cristina Castillo, Joaquín Hernandez, Juan Sotillo, Rodrigo Muiño, Jose L. Benedito, Ana Montes, Rafael Arana, Marta Matas-Quintanilla, Cándido G. Panizo, Ana María Gutiérrez Montes

**Affiliations:** 1 Department of Animal Pathology, Veterinary School, University of Santiago de Compostela, 27002 Lugo, Galicia Spain; 2 BioVetMed Research Group, Department of Animal Medicine and Surgery, Veterinary School, CEIR Campus Mare Nostrum (CMN), University of Murcia, 30100 Espinardo Murcia, Spain; 3 Animal Pathology Research Group Department of Animal Medicine and Surgery, Veterinary School, CEIR Campus Mare Nostrum (CMN), University of Murcia, 30100 Espinardo Murcia, Spain

**Keywords:** ADA, Saliva, Redox balance, Dairy cows, Early lactation

## Abstract

Enzyme adenosine deaminase (ADA) is a marker of inflammation in domestic animals, but it is unclear whether it is a reliable marker of oxidative stress, especially in the transition period in dairy cows. This study aims to assess if ADA and redox status measurements in saliva provide the same utility to detect disease condition as that obtained from serum. Sixty-eight multiparous Holstein cows, between 2 and 3 weeks postpartum were selected. Five study groups were established: control (healthy), and cows with ketosis, mastitis, laminitis, and metritis. The parameters measured were ADA activity, total oxidants (TOS), antioxidants (TAC), and OSi ratio.

Regarding redox status, no significant differences arise in both saliva and serum being the correlations negative and not significant. In saliva, ADA activity in healthy cows differs from those with pathological processes, having the lowest activities. In serum, ADA activity is similar in the healthy and ketosis cows, showing the lowest activities meanwhile animals with mastitis, laminitis, or metritis have significantly higher activities. In conclusion, the measurement of ADA activities and redox status in saliva does not give consistent results, being preferable to measure them in serum during the transition period.

## Introduction

High-producing dairy cows are predisposed to sickness at any time, especially after calving and during early lactation. These pathological processes are attributable to numerous causes: metabolic imbalances, impaired immune response, or even metabolic stress (Abuelo et al. 2019). Metritis (Rizzo et al. 2012), infertility (Shi et al. 2014), mastitis (Laliotis et al. 2020), and laminitis (Zhao et al. 2014) and could be connected, as a risk factor, with an imbalance in the oxidant/antioxidant ratio in early lactation, being these diseases, the most common cause of the economic losses suffered by dairy farms. Treatments for these diseases are often expensive, ineffective, or unprofitable. Therefore, an early diagnosis can help to reduce the incidence of these diseases (Hailemarian et al. 2014, Hernandez et al. 2020).

Metabolic stress in transition cows increases inflammation by constantly activating redox-sensitive transcription factors such as NFkB (Nuclear factor kappa-light-chain-enhancer of activated B cells) which leads to an increased expression of pro-inflammatory mediators that can cause damage to host tissues (Sordillo and Raphael 2013).

From a biological dimension, inflammation, which involves the release of chemokines and cytokines; dilation of blood vessels; and infiltration of immune cells, is the first-line immune response of an organism confronted with microbial infection or tissue injury (Hernandez et al. 2022). Metabolic stress can disrupt appropriate inflammatory responses, but there is sufficient evidence to suggest that inflammation can contribute to metabolic disorders as well (Cao et al., 2000).

Metabolically stressed cows are also known to produce excessive amounts of reactive oxygen species (ROS), which can damage the cells involved in the inflammatory response (Sordillo and Maravinga 2014, Abuelo et al. 2013).

So far, recognizing whether a dairy cow is under a metabolic stress state with a marked imbalance between oxidant and antioxidant substances has involved the measurement of blood specific parameters, such as total oxidant (Abuelo et al. 2019) and total antioxidant (Hernandez et al. 2022; Sanchez et al. 2019), and the ratio between both parameters, also called redox status, obtained through the OSi ratio (TOS/TAC) (Abuelo et al. 2013). Thus, an increase in this ratio indicates a risk of oxidative stress (OS) due to the increase in oxidant production and/or defensive antioxidant consumption (Sordillo and Maravinga 2014).

In this scenario and bearing in mind the relevance of the adenosine deaminase (ADA, EC 3.5.4.4) as a biomarker of immune and redox status in monogastrics (Mills et al. 2012; Sanchez et al. 2019), we consider that it is interesting to assess its usefulness in cattle. ADA is a ubiquitously expressed enzyme that can be found in several tissues and fluids and mediates the conversion of adenosine into inosine and of deoxyadenosine into deoxyinosine, playing a role in purine and pyrimidine metabolism (Sögüt et al. 2002), and participating an important role in the differentiation of B and T lymphocytes, as well as in maturation from monocyte to macrophage (Contreras- Aguilar et al. 2019), connected with immune function. Interestingly, this species only has the ADA-1 isoenzyme (Contreras- Aguilar et al. 2020), whereas in most species two isoenzymes have been detected: ADA-1 and ADA-2 (Sanchez et al. 2019). To the authors’ knowledge, few studies have addressed the usefulness of this salivary biomarker in dairy cows in comparison with those used to define oxidative stress (TOS and TAC) during early lactation. Many of them are related to the stimulation of the immune system (Sordillo and Aitken 2009) through the activation of inflammasomes which trigger a hyperinflammatory condition (Castillo et al. 2019) that can be reflected in changes in the ADA activities.

Recent studies have proposed using saliva instead of blood to test redox balance as it is a non-invasive method and because of its proved clinical usefulness as a biomarker of immune and redox status in monogastric. It is worth noting that in the case of ruminants the data available is limited. This may be because saliva production in these species is continuous throughout the day, due to their physiology, with a marked balance between chewing and resting times (Beauchemin et al. 2018).

Therefore, this study aims to find out whether measurements of ADA and redox status in saliva are useful in providing the same accurate information as that obtained from serum measurements of both parameters during the transition period of lactating cows.

## Materials and methods

### Animals and experimental procedure

Sixty-eight multiparous Holstein cows were used in this study. The cows were kept in three different barns located in Meira (Galicia, north-western Spain).

All the animals were kept under the same nutritional and management conditions throughout the study. They were kept using free-stall barns with concrete stalls and were fed a total mixed ration (TMR) formulated according to the National Research Council (2001) to meet their lactation. The diet before parturition comprised 13 kg of grass silage, 10 kg of maize silage, 3 kg of dehydrated alfalfa and 3 kg of a commercial concentrate composed of cereal grains (maize, wheat and barley) and oilseeds (soybean and cotton). Wheat straw (1-1.5 kg) was offered ad libitum. Chemical analyses of the TMR showed that the dry matter (DM as fed) content was 14.3 kg, containing 141 g of crude protein (CP)/kg of DM, and 1,5 Mcal/kg of net energy for lactation (NEL). The total mineral and vitamin composition of the diet was 12 mg/kg of Cu;18 mg/kg of Fe; 0.9 mg/kg of I; 54 mg/kg of Zn; 36 mg/kg of Mn; 0.180 mg/kg of Co; 0.27 mg/kg of Se; 7.200 IU vitamin A/kg, 2250 IU vitamin D3/kg, and 30 IU vitamin E/kg. The commercial concentrate had 18.8% CP content and 1.66 net energy lactation (NEL Mcal/kg). After calving, the diet was adjusted to maintain the requirements for milk output by increasing the amount of concentrate (2 kg more for a mean milk yield of 20 kg/d; 4 kg more for 25 kg/d; 6 kg for 30 kg/d and 7 kg for 35 kg/d). The cows were allowed to eat twice a day, in the morning from 08:00 to 11:00 and in the afternoon from 18:00 to 21:00. Cows were milked twice daily at 09:00 a.m. and 07:00 p.m.

The main selection criterion was that the cows were between 2 and 3 weeks postpartum, with no obstetric problems at calving. Other criteria were parity (entering their 2nd lactation or higher), milk production in the previous lactation (from 9.000 to 9.500 kg), and body condition score (from 3 to 3.5, on a scale from 1 [lean] to 5 [obese] according to the criteria established by Edmonson et al. (1989)

Once selected, the health status of the animals, as well as the diagnosis of the pathological processes (ketosis, laminitis, metritis, and mastitis), were determined by the same experienced veterinarian practitioner and according to the exploratory and diagnostic procedures outlined (Smith et al. 2020), albeit its results are not the objective of this study. After that, the cows were classified into different groups depending on whether they developed a postpartum and early lactation within the clinical parameters that they can be considered normal (or healthy) or if they had pathologies associated with redox imbalances (Abuelo et al. 2019) such as the existence of ketosis due to negative energy balance (NEB) (presence of ketonuria), laminitis (presence of lameness and digital dermatitis), mastitis (positive California Mastitis Test), and metritis (presence of purulent or mucopurulent uterine discharge with fever). Consequently, based on the clinical findings on the three barns, we established 5 study groups: healthy animals (CTRL Controls, n = 10); animals with ketosis (NEB, n = 17); animals with laminitis (LAM, n = 15); animals with mastitis (MAS, n = 13) and finally animals with metritis (MET, n = 13).

### Sample collection

Saliva samples were collected by allowing the cows to chew a piece of sponge approximately 1 cm x 1 cm in size, clipped to a thin metal rod, until completely moistened measuring for 1–2 min as previously reported (Contreras-Aguilar et al. 2020). Afterwards, the sponges were placed into specific tubes (Salivette tubes, SARSTEDT AG & Co. Germany) for centrifugation at 3000 g during 10 min.

All blood samples were taken at the same time, after the first meal, between 15:00 and 16:00, by jugular venipuncture. All tubes of blood were immediately placed on ice and centrifuged at 2500 g for 10 min at 4 ºC within 2 h after collection. Saliva and serum samples were collected from February to April and were stored at − 80 ºC until analysis.

Permission for the procedures of the experiment was granted by the Bioethical Committee of the University of Murcia (UMU) according to the Spanish Regulations (RD 53/2013, legal provision no. 1337), and the European Regulation of Animals for Scientific Purposes (Council of Europe, ETS no.123) (2010).

### Analytical procedures

#### Adenosine deaminase activity measurement

The ADA activity (ADAT) in the saliva and serum samples of cows was quantified, in duplicate, using a microplate adaptation of a commercial automatized human assay (ADA Biosystems S.A., Spain). The assay was optimised for both body fluids (see supplementary material). In the case of the saliva, it was necessary to use dilutions of 1:18 for the samples. However, in the case of the serum, no dilutions were necessary. The absorbance of a mixture consisting of 50 µL of the sample and 200 µL of ADA reagent was monitored for 3 min at 340 nm, and the ADA activity was obtained by calculating the maximal increase in absorbance per minute (U/L in the applied sample = Δabs/min * 3333). The intra-assay coefficient of variation of the assay was lower than 11%, and the limit of detection was 9.3 U/L.

#### Total antioxidant and oxidant capacity levels quantification

The quantification of the antioxidant status in saliva and serum samples of the cows, in duplicate, was performed using the ferric reducing antioxidant power (FRAP) assay. The coefficients of variation were below 9% for both intra and inter-assay precision and the limit of detection was 0.80 µM Trolox equivalents/L. In this case, no previous dilution of the samples was performed for any of the body fluids.

For the measurement of the oxidant status, in duplicate, a commercially available assay was optimised and validated (PierceTM Quantitative Peroxide assay, Thermo Scientific, United States). The calibration curve was constructed with peroxide in a range of 62.5 to 0.97 µM. The optimised assay for saliva samples consisted in the reaction of 120 µL of the sample with 100 µL of a working buffer, while the optimised assay for serum consisted in a reaction of 60 µL of the sample with 160 µL of a working buffer. The coefficients of variation were below 7% for the intra-assay precision and 10% for the inter-assay precision in both body fluids. The detection limit was 0.5 µM peroxidase equivalents/L.

The oxidative stress index (OSi) was calculated as total oxidant/ total antioxidant quotient (TOS/TAC) according to the studies conducted by Abuelo et al. (2013), which provide a global and objective assessment of the animal’s redox status. Although it is noteworthy to point out that it is not a matter to compare our numerical values with those described by Abuelo et al. (2013) because the parameters used to calculate the quotient are different from our study. Following such studies, an elevated OSi ratio compared to the healthy group indicates a risk of oxidative stress (OS) due to the increase in oxidant production and/or defensive antioxidant consumption.

### Statistical analysis

All statistical procedures were performed with IBM SPSS 25.0 for Windows. (IBM Co., Chicago, IL, USA). The data were checked for a normal distribution with the Kolmogorov-Smirnov test. When a parameter was non-normally distributed (all but TAC), a logarithmical transformation with subsequent verification was carried out using the abovementioned program. Once transformed to normal distribution all the non-normally distributed parameters, the data were analysed using a one-way analysis of variance (ANOVA test), including group as a fixed factor, and animal as a random factor. The means were compared using a Bonferroni test for post hoc analysis. Spearman correlation analysis was used to identify significant correlations between the oxidative status marker and ADA activities in both saliva and serum. The criterion for statistical significance was declared at P < 0.05.

## Results

**Table 1:** shows the mean values, together with the standard error of the mean (SEM) of salivary and serum TOS, TAC values OSi ratio and ADA activities

	Sample type			
^a^Parameters	^b^Group (N)	Saliva	Serum	Correlation coefficient (SL)
TOS values (µM/L peroxidase equivalent)	CTRL (10)	12.2 ± 1.2^a^	11.1 ± 0.9^a^	
	NEB (17)	11.2 ± 2.4^a^	8.3 ± 1.2^c^	
	LAM (15)	10.5 ± 0.9^a^	15.3 ± 4.8^a^	-0.022 (0.431)
	MAS (13)	12.3 ± 2.3^a^	6.9 ± 0.5^b^	
	MET (13)	10.8 ± 1.2^a^	6.0 ± 0.3^b^	
P-value		0.991	0.029	
TAC values (µM Trolox equivalent)	CTRL (10)	26.8 ± 6.4^a^	15.4 ± 1.4^a^	
	NEB (17)	24.8 ± 4.0^a^	16.0 ± 0.9^a^	
	LAM (15)	38.7 ± 7.6^a^	14.0 ± 1.2^a^	-0.077 (0.275)
	MAS (13)	27.9 ± 6.9^a^	11.6 ± 0.9^b^	
	MET (13)	30.7 ± 6.4^a^	12.4 ± 0.9^b^	
P-value		0.595	0.025	
OSi ratio (TOS/TAC)	CTRL (10)	0.74 ± 0.18^a^	0.75 ± 0.06^a^	
	NEB (17)	0.65 ± 0.16^a^	0.52 ± 0.06^a^	
	LAM (15)	0.7 ± 0.23^a^	1.21 ± 0.5^a^	-0.105 (0.206)
	MAS (13)	0.85 ± 0.2^a^	0.64 ± 0.07^a^	
	MET (13)	0.58 ± 0.21^a^	0.53 ± 0.07^a^	
P-value		0.993	0.121	
ADA Activities (IU/L)	CTRL (10)	386.4 ± 56.0^b^	235.2 ± 13.3^b^	
	NEB (17)	685.3 ± 93.8^c^	240.6 ± 12.6^b^	
	LAM (15)	642.2 ± 83.3^d^	292.5 ± 16.0^c^	0.335 (0.004)
	MAS (13)	1087.8 ± 96.7^a^	335.6 ± 20.8^a^	
	MET (13)	620.5 ± 78.9^e^	331.5 ± 31.7^a^	
P-value		0.001	0.001	

Table 1. Mean values (± standard error of the mean, SEM) of salivary and serum TOS, TAC, OSI ratio values and ADA activities and ^a^TOS: Total oxidant status; TAC: Total antioxidant capacity; OSi: Oxidative stress index; ADA: Enzyme adenosine deaminase type 1^; b^CTRL: Control group (healthy cows); NEB: Ketosis; LAM: Laminitis; MAS: Mastitis; MET: Metritis; ^c^SL: Significant level for correlation between parameters. N: number of samples measured.

The superscript letters (a, b, c…) within each column represent significantly different groups (P < 0.05). Different letters mean significant difference.

Note that the values of oxidants in saliva are statistically similar in CTRL and sick groups. However, the values of this parameter in serum show significant differences: TOS levels are higher in the LAM group, with slight statistical differences with respect to the CTRL cows. The lowest concentrations are recorded in the MAS and MET groups. The mean values in the NEB group appear as a group statistically different from the others. When establishing the level of correlation between the results depending on the different conditions and on the type of sample, it is found that this correlation is not significant.

When assessing the results obtained for antioxidant defence (TAC), we find the same trend. Salivary samples appear as a statistical group. When analyzing the serum data, the antioxidant defence is higher in the CTRL, NEB, and LAM groups, the three being statistically similar. The lowest levels appear in cows with mastitis (MAS) and metritis (MET) in a statistically different group. The correlation between the clinical condition and sample type is not significant.

Regarding the oxidant/antioxidant balance (OSi ratio), no significant differences arise in both types of specimens, with all situations acting as a single statistical group. Consequently, the correlations established are negative and no significant.

However, ADA enzyme activity shows significant differences for both types of samples. In saliva, ADA activity in the CTRL group differs statistically from the pathological diseases indicated, presenting the lowest values. In serum, ADA activity is statistically similar in the CTRL and NEB groups, with the lowest activities. Moreover, cows suffering from mastitis, laminitis, or metritis have significantly higher activities. The correlation between sample and clinical condition is also significant for this parameter.

**Fig. 1:**
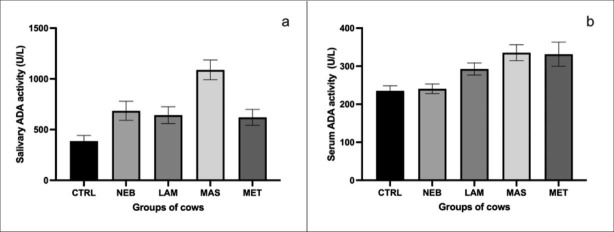
Mean and SEM values of ADA activity levels in saliva (a) and serum (b) samples of control cows (CRTL) and in cows with Ketosis (NEB), Lameness (LAM), Mastitis (MAS) and metritis (MET).

## Discussion

There are numerous studies in the literature discussing the role of ADA enzyme as a preventive biomarker of inflammatory disease in different species pointing out the differences between healthy and diseased animals. However, we must consider the fact that such many studies are due to several facts: the variety of techniques currently available to determine adenosine deaminase (ADA) enzyme activities and the diversity in the units of measurement given attributable to both the methodological diversity and the absence of a standard procedure (Contreras-Aguilar et al. 2019).

In general, our results agree with those described in numerous studies in which saliva (Contreras-Aguilar et al. 2019) and serum (Daliwal et al. 2020) ADA activities are higher in animals affected by infectious processes.

We can highlight previous studies (Fritzen et al. 2016; Fávero et al. 2018) that evaluate the changes manifested by this parameter in serum, throughout bovine pregnancy and in combination with those changes that occur in redox balance. These studies show that ADA values increase after parturition and that they are the consequence of the metabolic stress widely described (Abuelo et al. 2019; Contreras-Aguilar et al. 2020). In our study, serum ADA activities (see Fig. 1) increase in situations of laminitis, mastitis, or metritis, in line with the aforementioned studies (Abuelo et al. 2019, Lalilotis et al. 2020, Rizzo et al. 2012, Shi et al. 2014,).

The principle of the ADA assay is based on the detection of hydrogen peroxide or ammonia following the enzymatic deamination of adenosine to inosine by this enzyme. Dhaliwal et al. (2020) conclude that the ADA activity exerts a proinflammatory effect in the post-partum, contributing to the increase and decrease of pro and anti-inflammatory mediators respectively. Therefore, serum ADA acts in the immunomodulatory mechanism with protective effects during physiological conditions of compromised immunity such as calving. However, this principle does not apply to our case, as serum ADA activity is statistically the same for CTRL cows as for those with ketosis, it is similar for those with mastitis (MAS group) or metritis (MET group), but different for those with laminitis (LAM group). We think that, when analysing serum parameters, this measure is non-specific and lacks diagnostic value in this critical period. However, the increase in salivary ADA in the group of animals with ketosis should be further explored, since a more efficient detection of disease in saliva than in serum could explain the results as it has been reported in other species (Sánchez et al., 2021). Moreover, the different physiological effects of the adenosine are partly due to the fact that different adenosine receptors may have opposing functions so that the activation of one receptor may elicit a protective reaction on a second receptor or a pathological response on a third one. For the same reason, ADA can have pro or anti-inflammatory effects depending on the immune status (Fávero et al. 2018), so the role of ADA in ketosis should be clarified in further studies.

Although all the animals were kept under similar environmental, management, and dietary conditions, not all organisms act in the same way. Therefore, in the framework of our study, the results should be considered taking into account a clearly defined fact: the antioxidant status of an individual in such a complex situation depends largely on the individual endocrine changes that appear in early lactation and that are related to the distribution of nutrients for milk formation (Bagheri et al. 2019, Abuelo et al. 2019).

Considering this fact, when we look at cows with ketosis, we can see that TOS values are lower, whereas TAC values are not. This is probably because the body of these animals is synthesizing antioxidants to counteract the prooxidant load. This is understandable because in states of energy deficiency, alternative sources of energy are used to cope with lactation. The metabolization of such sources generates an excess of H+, and thus a situation of metabolic acidosis (Castillo et al. 2005). In this situation, the saliva, rich in bicarbonate, undergoes variations that are reflected in the changes in ADA activities. What is certain is that there is a lack of studies about the usefulness of salivary ADA as an early biomarker of metabolic imbalance.

Therefore, when dealing with the results relating to oxidative stress (TOS, TAC and OSI ratio) and ADA, currently, the measurement of redox status after calving must be made by assessing the oxidant/antioxidant profile and OSi ratio in serum, not in saliva, where the data are not conclusive. This fact may be attributable to changes in salivary secretion in cows throughout the day, which is conditioned by the structure of the diet received as well as by rumination cycles, which, in turn, vary among animals (Beauchemin et al. 2018, Maekawa et al. 2002). Therefore, we do not agree with the statement made by Contreras-Aguilar et al. (2019) concerning the fact that the measurement of salivary ADA activities may be an early marker of oxidative stress (Contreras-Aguilar et al. 2019), although it could differentiate postpartum pathological processes beyond a state of ketosis (NEB) that effectively can compromise the immune system (Castillo et al. 2006; Fritzen et al. 2016; Hernandez et al. 2022). Also, we have not found the correlation observed described by the same author between salivary ADA and inflammation in this species. Our data show that there are, effectively, significant differences both in saliva and in serum among healthy dairy cows and those affected by ketosis, LAM, MAS, and MET. This type of processes derives from the metabolic stress status in the transition period, which can only be confirmed by blood analysis, as can be observed in serum TOS and TAC (Sanchez et al. 2019) values and the OSi ratio, albeit no notable alterations in the Osi ratio of the saliva values are detected (Abuelo et al. 2013).

So, we could point out that a single parameter, like salivary ADA, in our opinion, cannot be a reliable reflection of redox status. In fact, when assessing these serum parameters, we note how, for each pathological situation, the serum values of oxidants and antioxidants are modified, changing, in turn, the OSi ratio (Abuelo et al. 2013), which shows no significant differences between diseases. This lack of significance may be the result of the individual action of each organism against the oxidative load that appears after parturition, and which has already been described in previous studies (Baumgard et al. 2017).

It should not be ignored that the development of a ketosis state in post-partum dairy cows is the beginning of a cascade of non-septic inflammatory processes that immunosuppress the animal (Abuelo et al. 2019, Cao et al. 2000, Castillo et al. 2005, Maekawa et al. 2002). We also point out that, in the period in which the study has been developed, salivary ADA activities above the range 330–442 IU/L in dairy cows - the range found in the CTRL (or healthy cows) group - could be considered as warning signal to undertake thorough monitoring of the affected animal, especially when the values reach the maximum, which correspond to the cases of mastitis.

It is important to mention that most studies in dairy cows were developed at a precise time, after the diagnosis of the disease (mastitis, laminitis, parasitosis, viral or bacterial infections, etc.). Their results indicate that the activities of salivary ADAs can be used to control the evolution of the disease and its treatment. However, we consider that, in order to know if we are at risk of internal imbalance or inflammation, further studies on this salivary biomarker should be performed, but not simply evaluating changes during different physiological stages such as growth or gestation or processes such as lactation as a function depending on the breed, diet or even production level.

The peculiarity of our study with respect to the data recorded in other studies, related to ADA activity, in cattle with similar processes (mastitis or laminitis) is the time chosen for the assessment, as in our study it widely exceeds the post-partum stage, and even the peak lactation (Frtizen et al. 2016, Sordillo and Maravinga 2014).

## Conclusion

Taking into account the conditions and analytical techniques used in our study, and given that this is the first study carried out in post-partum dairy cattle which analyses the ADA activities and its relationship with the redox balance measured in both saliva and serum, we can conclude that the measurement of ADA activities and redox status in saliva does not give consistent results, so it is recommended to assess them in serum, and in addition, ADA enzyme, except in the case of ketosis, is not shown to be an effective predictive marker of inflammation in cattle during the transition period.

## Data Availability

The data presented in this study are available on request from the corresponding author. This study complied with Spanish Regulations on animal experimentation (RD 53/2013, legal provision number 1337) and the European regulation of animals for scientific purposes (Council of Europe, ETS no.123).

## Abbreviations

ADA: Adenosine deaminase
CTRL: Control
NEB: Negative energy balance
LAM: Laminitis
MAS: Mastitis
MET: Metritis
TOS: Total oxidants
TAC: antioxidants
OSI: oxidant/antioxidant ratio
NFkB: Nuclear factor kappa-light-chain-enhancer of activated B cells
ROS: Reactive oxygen species
OS: Oxidative stress
TMR: Total mixed ration
DM: Dry matter
CP: Crude protein
NEL: Net energy for lactation
FRAP: ferric antioxidant power
SEM: Standard error of the mean.
